# ‘Strategic’ development of precision cancer medicine in the United States

**DOI:** 10.1002/1878-0261.13023

**Published:** 2021-06-24

**Authors:** Richard L. Schilsky

**Affiliations:** ^1^ University of Chicago Chicago IL USA

**Keywords:** cancer care delivery, cancer research funding, molecular profiling, precision oncology

## Abstract

The availability of advanced omics technologies has largely driven development of precision cancer medicine in the United States, but integration in routine cancer care has been challenging. Here, we consider some parameters that would enable a more coordinated, integrated, efficient, and equitable implementation of precision cancer medicine.

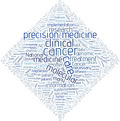

**Abbreviations**
ARPA‐H: Advanced Research Projects Agency for Health
CMS: Centers for Medicare and Medicaid Services
EHR: electronic health records
FDA: Food and Drug Administration
NCI: National Cancer Institute
NIH: National Institutes of Health
OHRP: Office for Human Research Protections

Oncologists have recognized for many years that cancer is not a single disease, but it has only been recently that the enormous biological diversity of cancer has been revealed through sophisticated molecular profiling studies of human tumors, particularly through genomic profiling. It is probable that no two cancers are alike in their genomic, transcriptomic, and proteomic profiles or microenvironment. This biological heterogeneity gives rise to cancers that vary in clinical presentation, natural history, prognosis and response to treatment. The clinical assessment of a patient's cancer is further complicated by the fact that cancers are spatially heterogeneous and evolve over time in response to treatment and attack by the host immune system. That is, different regions of any tumor nodule in a patient likely harbor different malignant clones that can be identified by unique genomic profiles. The tumor itself is not static but changes under the selection pressures of treatment such that clones of sensitive cells regress while those of resistant cells emerge giving rise to the familiar clinical scenario of initial response to treatment followed by disease progression and drug resistance.

Development of precision cancer medicine until now has been driven largely by the availability of DNA sequencing technologies that enable assessment of large numbers of genes in a single analysis and with a sufficiently rapid turnaround time to be applicable to clinical care. However, implementation of precision cancer medicine as part of routine cancer care is challenging in view of the enormous diversity of the cancer patient population, the need to coordinate care among the many specialists who comprise multi‐disciplinary cancer care teams, and, in the United States, the fragmented healthcare delivery system that may limit access to care and sharing of information among specialists. This new paradigm of cancer care also requires that oncologists acquire or have ready access to information about the genomics of cancer and the molecular pharmacology of targeted cancer drugs to supplement their clinical knowledge of the natural history of each cancer type and their assessment of each patient. Detailed and validated standard operating procedures are necessary to ensure quality in every step of the precision medicine workflow from specimen acquisition and handling to biomarker detection and quantification to physician interpretation and treatment planning. Increasingly, oncology professionals are challenged to recognize the molecular subsets of common cancers, interpret results of complex molecular diagnostic tests, develop appropriate treatment plans, and deliver state‐of‐the‐art care when clinical guidelines and clinical decision support services are either lacking or outdated.

In the United States, the emergence of precision cancer medicine is occurring in the context of a healthcare system that is plagued by fragmented care delivery, siloed medical information, variable insurance coverage, and inequities in access to care across the population. Even with the advent and broad adoption of electronic health records (EHR), complete clinical information about a patient is not always integrated into a single clinical record as some information may reside in separate pathology, radiology, or pharmacy record systems that do not interface easily with the primary EHR. This is often the case for genomic test reports that are increasingly necessary for optimal clinical care. The result is often poor information exchange among providers that leads to repetitive and unnecessary testing, polypharmacy, and treatment plans that are not developed, communicated, or applied in a coordinated way resulting in excessive expenditures and administrative burdens. Despite the investment of enormous resources, cancer care and research in the United States is best described as decentralized, fragmented, and market‐driven with few national standards, limited data integration and sharing, and lack of a coordinated national strategy to established research priorities. Indeed, the U.S. precision cancer medicine enterprise is a patchwork of Federal agencies that support research [National Institutes of Health (NIH), National Cancer Institute (NCI)], oversee the conduct of research [NIH, NCI, Food and Drug Administration (FDA), Office for Human Research Protections], regulate and reimburse the delivery of care (FDA and Centers for Medicare and Medicaid Services (CMS), and capture real‐world experience through registries (Centers for Disease Control and Prevention, NCI, CMS). Other key entities include the NCI‐designated cancer centers, the NCI‐sponsored National Clinical Trials Network, professional societies, and guideline bodies and payers all of which can impact who gets care, what care they receive, where they receive it, and how it is paid for.

As specified in the National Cancer Act of 1971, leadership of the National Cancer Program nominally rests with the Director of the NCI who is authorized to formulate an annual plan and budget proposal that is submitted directly to the President. For the fiscal year 2022, the current NCI Director has proposed a budget of $7.415 billion to focus on priority areas of cancer drug resistance, molecular diagnostics for cancer treatment, obesity and cancer, and cancer survivorship [1]. The NCI also has funds available from the Cancer Moonshot, approved by Congress in 2016, and has invested more than $1 billion thus far in over 70 consortia or research programs and more than 240 research projects in high‐priority areas including cancer immunotherapy, advancing childhood cancer research, expanding cancer prevention, and early detection and mapping tumors [2]. Recently, the US President Joe Biden called for creation of an Advanced Research Projects Agency for Health (ARPA‐H) to speed development of high‐risk projects to advance progress against cancer, diabetes, and Alzheimer's disease. A budget of $6.5 billion has been proposed for the fiscal year 2022 in support of this new agency, although its exact functions and leadership have not been announced at this time [3]. The American Society of Clinical Oncology also publishes annual cancer research priorities as part of its annual report on *Clinical Cancer Advances* [4].

As alluded to previously, implementation of precision cancer medicine in a healthcare system requires substantial planning and considerable investment. Major considerations include the following:

- Molecular analysis of tumor specimens: use a commercial laboratory *vs*. build a certified ’omics laboratory?
- Building/curating the knowledge base necessary for the analysis and reporting of ‘omics data.
- Assembling interdisciplinary teams of clinicians, bioinformaticians, and molecular pathologists to interpret results from complex molecular diagnostic tests and link them to clinical profiles and treatment recommendations.
- Obtaining and analyzing clinical outcomes to validate utility of the various treatment options.
- Delivering provider decision support and patient education.
- Investing in genetic counselors necessary to help patients understand and make informed decisions about their care.
- The storage, management, and sharing of ‘omics data involve major computational resources, data security requirements, and, in some circumstances, ethical challenges.
- The field is rapidly moving and requires continuous attention and updating by testing laboratories, molecular pathologists, clinical oncologists, regulators, and payers
- Who pays for implementing the infrastructure required to deliver precision cancer medicine? How is the return on those investments achieved and assessed?

While implementation of precision cancer medicine in the United States is often technologically far advanced, it is plagued by lack of standards, lack of quality measures, inconsistent regulatory policies, inequities in access to care, and challenges in gathering and reporting real‐world experience. Market forces and competition among institutions are key drivers of implementation of precision medicine programs. While some large commercial laboratories have obtained FDA approval of their genomic test platforms, many hospitals have established ‘in house’ molecular diagnostic laboratories that offer comprehensive molecular testing that has not been subjected to rigorous clinical validation or regulatory review. Clinical interpretation of molecular tests is left to clinicians, often assisted by institutional experts who sometimes convene as ‘molecular tumor boards’. The results of genomic tests are used to identify standard of care targeted therapies for patients, navigate patients to clinical trials of investigational agents, or propose off‐label treatments of potential, but often unproven, utility. The latter are often difficult for patients to access due to lack of insurance coverage and reimbursement, and when accessible, it is rare that treatment outcomes are recorded, captured in registries, and disseminated in scholarly publications that inform the community about the utility of such approaches.

The implementation of precision cancer medicine holds great promise but also continues to present many challenges that are related in large part to lack of standard definitions and data elements, poor specification of standard operating procedures, limited sharing of best practices, and poor annotation of clinical outcomes in the context of routine care. Due, in part, to the fragmented healthcare delivery system in the United States, risk exists that not all segments of the population will be able to access the sophisticated care teams and technologies necessary to deliver precision cancer care and that disparities in outcomes will heighten as a result.

To some extent, the current deployment of precision cancer care across the globe can be summarized by a statement by the American poet T.S. Eliot made in a different context and long before the advent of precision medicine, but still applicable [5]:

The vast accumulations of knowledge—or at least of information… have been responsible for an equally vast ignorance. When there is so much to be known, when there are so many fields of knowledge in which the same words are used with different meanings, when everyone knows a little about a great many things, it becomes increasingly difficult for anyone to know whether he knows what he is talking about or not…

As Europe looks to the future of precision cancer care, European scientists, clinicians, and policy‐makers can learn a great deal from observing the deployment of precision cancer medicine in the United States and should aim for a more coordinated, integrated, efficient, and equitable process.

## Conflict of interest

Dr. Schilsky serves as principal investigator of the ASCO TAPUR study. ASCO receives research grants from the following companies in support of the study: Astra‐Zeneca, Bayer, Boehringer‐Ingelheim, Bristol Myers Squibb, Genentech, Lilly, Merck, Pfizer, Seattle Genetics. Dr. Schilsky serves as a member of the managing board of Clariifi and as a consultant to Bryologyx, Cellworks Group, EQRx, and Scandion Oncology.
